# Apoptosis and inflammation

**DOI:** 10.1155/S0962935195000020

**Published:** 1995-01

**Authors:** C. Haanen, I. Vermes

**Affiliations:** 1 Department of Clinical Chemistry Medical Spectrum Twente P.O. Box 50.000 Enschede NL-7500 KA The Netherlands

## Abstract

During the last few decades it has been recognized that cell death is not the consequence of accidental injury, but is the expression of a cell suicide programme. Kerr et al. (1972) introduced the term apoptosis. This form of cell death is under the influence of hormones, growth factors and cytokines, which depending upon the receptors present on the target cells, may activate a genetically controlled cell elimination process. During apoptosis the cell membrane remains intact and the cell breaks into apoptotic bodies, which are phagocytosed. Apoptosis, in contrast to necrosis, is not harmful to the host and does not induce any inflammatory reaction. The principal event that leads to inflammatory disease is cell damage, induced by chemical/physical injury, anoxia or starvation. Cell damage means leakage of cell contents into the adjacent tissues, resulting in the capillary transmigration of granulocytes to the injured tissue. The accumulation of neutrophils and release of enzymes and oxygen radicals enhances the inflammatory reaction. Until now there has been little research into the factors controlling the accumulation and the tissue load of granulocytes and their histotoxic products in inflammatory processes. Neutrophil apoptosis may represent an important event in the control of intlamtnation. It has been assumed that granulocytes disintegrate to apoptotic bodies before their fragments are removed by local macrophages. Removal of neutrophils from the inflammatory site without release of granule contents is of paramount importance for cessation of inflammation. In conclusion, apoptotic cell death plays an important role in inflammatory processes and in the resolution of inflammatory reactions. The facts known at present should stimulate further research into the role of neutrophil, eosinophil and macrophage apoptosis in inflammatory diseases.


Mediators of Inflammation 4, 5–15 (1995)

### Apoptosis and inflammation

C. Haanen and I. Vermes[CA]

Department of Clinical Chemistry, Medical Spectrum Twente, P.O. Box 50.000, NL-7500 KA Enschede, The Netherlands

[CA] Corresponding Author

**DURING the last few decades it has been recognized that cell death is not the consequence of accidental injury, but is the expression of a cell suicide programme. Kerr *et al.* (1972) introduced the term apoptosis. This form of cell death is under the influence of hormones, growth factors and cytokines, which depending upon the receptors present on the target cells, may activate a genetically controlled cell elimination process. During apoptosis the cell membrane remains intact and the cell breaks into apoptotic bodies, which are phagocytosed. Apoptosis, in contrast to necrosis, is not harmful to the host and does not induce any inflammatory reaction. The principal event that leads to inflammatory disease is cell damage, induced by chemical/physical injury, anoxia or starvation. Cell damage means leakage of cell contents into the adjacent tissues, resulting in the capillary transmigration of granulocytes to the injured tissue. The accumulation of neutrophils and release of enzymes and oxygen radicals enhances the inflammatory reaction. Until now there has been little research into the factors controlling the accumulation and the tissue load of granulocytes and their histotoxic products in inflammatory processes. Neutrophil apoptosis may represent an important event in the control of inflammation. It has been assumed that granulocytes disintegrate to apoptotic bodies before their fragments are removed by local macrophages. Removal of neutrophils from the inflammatory site without release of granule contents is of paramount importance for cessation of inflammation. In conclusion, apoptotic cell death plays an important role in inflammatory processes and in the resolution of inflammatory reactions. The facts known at present should stimulate further research into the role of neutrophil, eosinophil and macrophage apoptosis in inflammatory diseases.**

**Key words:** Apoptosis, Cytokines, Growth factors, Hormones, Inflammation, Integrins.

### Introduction

During the last few decades it has been recognized that cell death is generally not the consequence of ageing or accidental injury, but that cells die as the expression of a genetically programmed active cell suicide. This physiological form of cell death, which is profoundly influenced by the extracellular microenvironment, is of fundamental significance in embryogenesis, morphogenesis, and in the processes that govern tissue shape and cell renewal. Active elimination of cells was observed originally in 1951 by Glücksmann[1] and in 1966 by Saunders[2] as an essential feature in the development of vertebrates and invertebrates, respectively. They recognized programmed cell death as a mechanism by which unwanted or useless cells are eliminated. In the past ten years it has become increasingly apparent that programmed cell death occurs not only during embryonic development, but appears to be a widespread phenomenon, and a dynamic balance between cell proliferation and cell elimination.[3,4] Programmed cell death is responsible for the deletion of useless, unwanted or crippled cells, remodelling of organs, canalization of ducts, fashioning of the body, formation of digits, and fusion of palatal shelves.

Necrosis has been the traditional term for accidental cell death. Necrosis occurs in response to harmful insults such as physical damage, hypoxia, hyperthermia, starvation, complement attack, and chemical injury. The earliest morphological changes that occur during necrosis, are swelling of the cytoplasm and organelles. These changes are the expression of the loss of selective permeability of the cytoplasmic membrane. This demolition results in dissolution of the organelles, leaking of the cellular contents into the extracellular space, and finally in the disappearance of the cytoplasmic membrane. Necrosis affects tissue areas or groups of contiguous cells and elicits an inflammatory reaction in the

© 1995 Rapid Communications of Oxford Ltd adjacent viable tissues in response to the released cell debris.

Apoptosis shows, morphologically, a distinct pattern of cell resolution. The earliest changes include the loss of cell junctions and specialized membrane structures such as microvilli. The cytoplasm shrinks and the nucleus coalesces into condensed masses, which later break up into fragments. The mitochondria initially remain intact. The endoplasmic reticulum transforms into vesicles that fuse with the cytoplasmic membrane. These processes result in contraction of the cytoplasmic volume, associated with the loss of intracellular fluid and ions. The cell adopts a convoluted outline and subsequently breaks into several membrane-enclosed apoptotic bodies containing well-preserved organelles and nuclear fragments. The apoptotic bodies are phagocytosed and digested by nearby resident cells. The engulfing cells belong mostly to the mononuclear-phagocyte system, but epithelial or endothelial cells, and even tumour cells, may also be involved. It has been recognized that apoptotic bodies themselves provide a stimulus for phagocytosis by changes on their cytoplasmic membrane. Once ingested the apoptotic bodies undergo rapid degradation. Apoptosis does not induce any inflammatory reaction. A characteristic biochemical feature of the process is double-strand cleavage of nuclear DNA at the linker regions between nucleosomes, leading to the production of oligonucleosomal fragments of ± 200 kDa or multiples of it. Apoptosis is not harmful to the host and is in fact necessary for a normal existence. Distinctive morphological and biochemical characteristics have been reviewed by Wyllie *et al.*[3] and Trump *et al.*[5]

In 1972 Kerr *et al.* described the ultrastructural and morphological differences that can be observed between necrosis and programmed cell death[6]. They introduced the term apoptosis as opposite to that of necrosis. Apoptosis is derived from the Greek term αποπτωσις that indicates the fall of leaves from trees or the shedding of petals from flowers.

At present, the term apoptosis is often used synonymously with programmed cell death. The melding of the two concepts may lead to confusion. Programmed cell death is the definition of a 'concerted programme' of cell elimination during metamorphosis, embryogenesis and morphogenesis. Apoptosis indicates the phenomenon of 'physiological cell death,' which does not include any programming.

Apoptosis takes place during embryogenesis, during the development of the nervous system and the immune system, in the course of normal tissue turn over, and after withdrawal of trophic hormones or cytokines from target tissues. Apoptosis is implicated in the homeostatic cell balance in normal adult tissues such as intestinal crypts, the skin, the haematopoietic system. Neutrophils undergo apoptosis during resolution of the inflammatory reaction. Apoptosis of lymphocytes is an essential mechanism in the regulation of the immune system. It occurs during evolution of hormone dependent organs (prostate, endometrium, mammary tissue). Cell injury due to a variety of agents (viral infection, physical injury, chemical insults) may also lead to apoptosis. In general, any event that can produce necrosis by cell destruction (toxins, radiation) can induce apoptosis if the cell initially survives. Finally apoptosis has been demonstrated in premalignant and malignant tissues. Comprehensive reviews have recently been given by Ellis *et al.*,[7] Alison and Saraf,[8] Cohen,[9] Schwartzman and Cidlowski,[10] Gewitz,[11] and Vermes and Haanen.[12]

The most prominent morphological features and biochemical differences between apoptosis and necrosis are summarized in Table 1. This review covers present knowledge about mediators and modulators of apoptosis, and the biochemical mechanisms and its genetic control. Special emphasis is given to the influence of hormones, cytokines and growth factors in the occurrence of apoptosis and to the role of apoptosis in inflammatory disease.

### Mediators/Modulators of Apoptosis

Apoptosis is induced by stimuli that trigger intracellular responses that result in a characteristic type of cell death. This concept implies the existence of specific stimuli and the presence of target cells. An important characteristic of apoptosis is that it is induced by withdrawal of cell specific stimuli and can be inhibited by the action of cell specific hormones, growth factors and mitogens. We may speculate about the advantages of cell survival, depending upon signals produced by other cells. One possibility is that it could provide a mechanism for eliminating cells that end up in an abnormal location. For example, when a tissue is lacerated, cells may become displaced or transported by the circulation into places where they could cause trouble if they survived and proliferated. The same mechanism may prevent haematopoietic stem cells from proliferating outside the bone marrow, or cancer cells from establishing metastases. Another advantage of cell survival being independent of the presence of specific growth factors, is that control of the total number of distinct cell types, because they have to compete for limiting amounts of growth factors, establishes a continuous selection for the most competitive and vital cells.

*Hormones:* In mammals apoptosis is prominent in certain tissues undergoing atrophy as a result of withdrawal of hormones. It is observed during tissue involution, for example in the uterus after delivery

**Table 1.** Morphological and biochemical differences between apoptosis and necrosis

| Features | Apoptosis | Necrosis |
|---|---|---|
| Stimuli | Physiological | Pathological |
| Origin | Lack of growth factor<br>Hormonal influence<br>Mild toxic stimulus | Anoxia, Starvation<br>Physical insult<br>Chemical injury |
| Occult phase | Minutes to hours | None |
| Adhesion property | Immediately lost | Initially intact |
| First manifestation | Cell shrinking | Cell swelling |
| Nuclear changes | Condensation, karyorrhexis | Karyolysis |
| Nuclear chromatin | Margination, segmentation | Nuclear folding |
| Nucleolar changes | Intact, later degraded | Granulated |
| Membrane integrity | Persists for some time | Early failure |
| Surface morphology | Smoothing, blebbing | Lysis |
| Surface changes | Expression vitronectin<br>thrombospondin | None<br>None |
| Cytoskeletal changes | Surface protrusions<br>Cytoplasmic budding<br>Formation of apoptotic bodies | Fragmentation<br>Leakage of cellular contents |
| Mitochondria | Initially unaffected | Swelling, $Ca^{2+}$ uptake |
| ER/Golgi apparatus | Initially unaffected | Dilated |
| Organelles | Structurally intact | Swollen, leaky |
| Protein synthesis | Process can be blocked by actinomycin D, cycloheximide | Not affected by antibiotics |
| Cytoplasmatic changes | $Ca^{2+}$ ↑, endonuclease ↑<br>transglutaminase ↑<br>p53 ↑, *bcl-2* ↓, *c-myc* ↑ | Rupture lysosomes<br>Release of content |
| Nuclear changes | Internucleosome cleavage<br>DNA laddering | Diffuse degradation<br>DNA smear |
| Cells affected | Individual cells<br>Scattered cells | Groups of contiguous cells, tissue areas |
| Cell elimination | Engulfment by macrophages and endothelial cells | Exudative inflammation in adjacent tissues |
| Scar formation | Absent | Present |

and in the breast gland after weaning. Hypophysectomy induces apoptosis in the adrenal cortex and in the thyroid gland.[3] Apoptosis occurs in endometrial cells deprived of steroid hormones[13,14] and in the prostatic glandular acini after orchidectomy.[15] While some hormones prevent apoptotic death of susceptible cells, others trigger apoptosis; for example, corticosteroids in thymocytes and lymphocytes (see Table 2).[3,16] The mechanism of lymphocyte killing by glucocorticoids is unclear. Transcriptional regulation is implicated since an intact transcriptional transactivation domain of the glucocorticoid receptor gene is required.[17] In all these examples the signals that either trigger or prevent apoptosis also control other aspects of growth and development of susceptible cells. In other words both cell replication and cell death are regulated in concert to produce growth and cell regression, and to balance the size of organs.

*Growth factors:* It has been suggested that all animal cells are dependent on the presence of at least one survival factor.[18] These survival factors include growth factors. The nerve growth factor (NGF) was the first growth factor to be identified and characterized. Neurons die if they receive inadequate neurotrophic support; conversely neuronal death is prevented when exogenous NGF is provided.[19,20] There is evidence that the targets of neurons may not be the only source of NGF and that other peptides may act as a survival signal for neurons and glial cells. Such peptides include platelet derived growth factor (PDGF), insulin-like growth factor (IGF), fibroblast growth factor (FGF) and ciliary neurotrophic factor (CNTF).[21–25] Cells that are in cycle are much more dependent upon the presence of growth factors, than are non-cycling cells. Fibroblasts engineered to express *c-myc* and which therefore constitutively remain in cycle, undergo apoptosis when deprived of a restricted group of cytokines, principally IGF an PDGF.[26] The death of haematopoietic cells, such as normoblasts, neutrophils and megakaryocytes, has the morphological and biochemical characteristics of apoptosis. The suppression of apoptotic cell death of haematopoietic (precursor) cells requires the continuous presence of haematopoietic growth factors such as erythropoietin (EPO) and colony stimulating factors (CSFs).[27] Next to CSFs, IGF also prevents the occurrence of apoptosis in IL-3 dependent haematopoietic cells.[28] Apoptosis of erythroid progenitor cells is suppressed by erythropoietin (EPO).[29,30] Williams *et al.*[27] observed that occurrence of apoptotic death of myelo-monocytic progenitor

**Table 2.** Enhancement or suppression of apoptosis by tissue specific hormones, growth factors and cytokines

| Cells/tissues | | Apoptosis | |
|---|---|---|---|
| | | Enhance | Suppress |
| | Hormones | | |
| Adrenal cortex | Adrenocorticotropic hormone | | + |
| Prostrate | Testosterone | | + |
| Endometrium | Oestrogen hormone | | + |
| | Progesterone | | + |
| Thymocytes | Corticoids | + | |
| | Growth factors | | |
| Neurons | NGF, PDGF, IGF, FGF, CNTF | | + |
| Primordial germ cells | SCF | | + |
| Endothelial cells | FGF | | + |
| Haemopoietic cells | IGF | | + |
| Myeloid progenitor cells | CSFs, G-CSF, GM-CFS | | + |
| Neutrophils | NGF | | + |
| Mast cells | IGF-1 | | + |
| 7-M12 leukaemia cell line | TGF-β1 | + | |
| Tumour cells | Gfs (autocine production) | | + |
| | Cytokines | | |
| Neurons | LIF | + | |
| Haemopoietic(precursor)cells | IL-3 | | + |
| Activated precursor cells | TNFα | + | |
| Neutrophils | IL-3 | | + |
| | TNFα | + | |
| Eosinophils | IL-5 | | + |
| Mast cells | IL-3 | | + |
| Monocytes/macrophages | IL-1β, TNFα | | + |
| Stimulated monocytes/ | IL-4 | + | |
| macrophages | IFNγ | | + |
| Thymocytes | TNF | + | |
| T-lymphocytes | IL-2, IL-4 | | + |
| CD4+/CD8+ T-lymphocytes | IL-2 | + | |
| Megakaryoblasts | IL-11 | | + |
| B-chron. lymph. leukaemia | IL-4 | | + |
| blc2 + chronic lymphatic leukaemia | IL-4 | | + |
| 7-M12 leukaemia cell line | IL-3, IL-6 | | + |
| Tumour cells | Cytokines (autocrine production) | | + |

Abbreviations: CNTF, ciliary neutrophic factor; CSF, colony stimulating factor; G-CSF, granulocyte CSF; GM-CSF, granulocyte/monocyte CSF; FGF, fibroblast growth factor; IFN, interferon; IL, interleukin; IGF, insulin like growth factor; LIF, leukaemia inhibiting factor; NGF, nerve growth factor; PDGF, platelet derived growth factor; TGF, transforming growth factor; TNF, tumour necrosis factor; SCF, stem cell factor.

cells was suppressed when either granulocyte colony stimulating factor (G-CSF) or granulocyte–macrophage colony stimulating factor (GM-CSF) was added to the culture medium. Just like the granulocyte–macrophage precursors, which are lost during culturing unless exposed to GM-CSF or G-CSF, the survival of neutrophils is dependent upon the continuous presence of these factors.[31,32] Recently it has been described that nerve growth factor (NGF) has an inhibitory effect on the occurrence of apoptosis in murine peritoneal neutrophils.[33] Endothelial cells in culture undergo apoptosis when deprived of FGF.[34] A mechanism by which various tumour cells evade apoptosis is by the autocrine production of various survival factors, such as IGF or cytokines.

In conclusion, as indicated in Table 2, various types of growth factors, such as CSFs, FGF, PDGF, NGF and IGF inhibit apoptosis of susceptible cells.

*Cytokines:* Not only growth factors control cell proliferation and maturation, for cytokines also modulate the viability of the cells. Apoptosis of sympathetic neurons is inhibited by NGF, but is enhanced by leukaemia inhibitory factor (LIF).[35] The same mechanism applies for cycling haematopoietic progenitor cells, which undergo apoptosis under the influence of tumour necrosis factor-α(TNFα),[36] or when certain cytokines (e.g. IL-3) are removed.[37,38] Many authors have shown that apoptotic death of haemopoietic progenitor cells is prevented by addition of interleukin-3 (IL-3).[39–43] The cytokine IL-3 regulates the development and differentiation of neutrophils in concert with CSFs.[31–42] Erythropoietin (EPO) retards DNA breakdown and prevents apoptosis in erythroid progenitor cells.[44] The haematopoietic cytokines EPO, IL-3 and stem cell factor (SCF) are necessary during the mid-gestation haematopoietic development in the foetal mouse liver to protect these cells from apoptosis in liver during ontogeny.[45] Mature neutrophils undergo apoptosis on ageing,[46,47] a process that is enhanced by tumour necrosis factor-α(TNFα).[36] TNF also enhances cAMP induced apoptosis in mouse thymocytes[48,49] and in the human histiocytic lymphoma cell line U937.[50] TNF secreted by lymphocytes and macrophages, triggers apoptosis in many cell types. Apoptosis induced in the murine leukaemia cell line 7-M12 by transforming growth factor (TGF-β1) or cytotoxic agents, can be counteracted by IL-3 and IL-6.[51]

Mature T lymphocytes undergo apoptosis when deprived of IL-2,[52,53] whereas IL-2 primes CD4 + and CD8+ mouse T lymphocytes to undergo apoptosis when, subsequently, they are exposed to antigen which triggers the TCR.[54,55] IL-2 and IL-4 selectively rescue T cell subsets from glucocorticoid-induced apoptosis.[56] IL-4 inhibits apoptotic cell death in B-cell chronic lymphocytic leukaemia cells *in vitro*.[57]

The rate of apoptosis of human eosinophils, which is slower than that reported for neutrophils, is delayed *in vitro* by the eosinophil differentiation factor IL-5, which appears to have no effect on neutrophil apoptosis.[24,58,59] Therefore, despite the close haematopoietic origin of eosinophils and neutrophils, apoptosis and longevity, like their growth and differentiation, are controlled by different specific growth factors and cytokines.

During apoptosis of monocytes/macrophages IL-1 is released, which binds with high affinity to specific receptors on target cells.[60] TNFα and IL-1β prevent apoptotic death in human monocytes/ macrophages.[61] Apoptosis of stimulated monocytes/ macrophages is inhibited by IFN-γ (from TH-1 cells and enhanced by IL-4 (from TH-2 cells).[62] Murine mast cells undergo apoptosis on removal of IL-3.[63] Human megakaryoblastic cell lines produce IL-11, which acts as an autocrine growth factor in these cells.[64]

In conclusion, as summarized in Table 2, cytokines released by lymphocytes, monocytes or macrophages, which bind to cell receptors, modulate the viability and occurrence of apoptotic death in susceptible cells.

*Killer cells/cytotoxic lymphocytes:* During the maturation of T-lymphocytes in the thymus, a rearrangement takes place in the T-cell receptor genes, which results in the production of T cells with a tremendous variety of T-cell receptors (TCRs). This random process results in many T cells that bear TCRs and can react against components of their own tissues. Self-reactive T cells are potentially dangerous and must be eliminated before they enter the circulation. In the thymus the T cells that react with self-antigens undergo apoptosis, a negative selection procedure. T cells must not only learn to avoid attacking self-antigens, they must also learn to detect their targets only when encountered as a peptide presented as localized in the groove of the major histocompatibility complex (MHC) on the surface of an antigen-presenting cell. This is a positive selection procedure, known as MHC restriction. Consequently, immature T cells that bind to self-antigens or to antigens not presented in association with the MHC complex are eliminated by apoptosis.[65–67] T cells which survive the selection procedure go on to reach maturity and leave the thymus.

The MHC molecules consist of two types: (1) Class I MHC displays peptides from proteins made inside a cell (e.g. virus infected cells, cancer cells); and (2) Class II MHC displays peptides from proteins that have entered the cell from outside (e.g. foreign, abnormal, altered proteins, bacterial toxins).

T-lymphocytes occur in two varieties, depending upon the structure of a co-receptor next to TCR:

(1) T cells with co-receptor CD8+ recognize antigens only when displayed by Class I MHC and become cytotoxic lymphocytes (CTL) or natural killer (NK) cells. CTLs and NK cells recognize foreign antigens displayed by aberrant, malignant or virus-infected cells, which they then destroy by inducing apoptosis.

(2) T cells with co-receptor CD4+ recognize antigens only when presented by Class II MHC and become T helper cells (TH cells). TH cells produce interleukins (ILs); factors that spur other T or B cells into action, such as gamma interferon (IFNγ) ← TH-1 cells and IL-4 ← TH-2 cells.[68]

A CTL with a TCR that fits onto an MHC/antigen complex presented by a macrophage, brings IL-2 receptors to expression. In case of TCR activation under the influence of IL-2, the IL-2 receptors enhance the TCR/CD-3 signal to the nucleus to start CTL cell reduplication. Otherwise, when deprived of IL-2, the CTLs undergo apoptosis. After antigen clearance, IL-2 production ceases because TH cells are no longer stimulated and consequently the antigen responsive T cells undergo apoptosis. This mechanism has the physiological role of terminating the immune response and reducing the increased cell numbers to original proportions.

Unlike T cells, B cells secrete their receptors, which then circulate in the blood as antibodies. These antibodies lock onto antigens and help to destroy them. B cells express the same variety in antibody production as do the T cells in their TCRs. If antibodies present on B cells engage with an antigen, these cells are stimulated to proliferate and to secrete large amounts of the same kind of antibodies. One of the remarkable qualities of immunoglobulin production is that the affinity of the antibodies to antigen improves during the period of immune reaction. This is due to the fact that the B cells, which carry the most avidly binding antibodies, are stimulated to proliferate at the expense of those bearing the poorer antibodies, which are deleted by apoptosis.[69,70] Germinal centre B cells, which do not receive antigen stimulation of their surface immunoglobulin, undergo apoptosis.[71]

Comprehensive reviews about apoptosis in immunogenesis have been given by Golstein *et al.*,[72] Von Boehmer,[73] Cohen and Duke,[74] Fesus[75] and Kabelitz *et al.*[76] Summarizing, the immune system is 'taught' during its development and in further life, to distinguish between self and non-self and to improve the quality of the immune response, where apoptosis is their 'teacher'.

*Cell receptors:* There are a number of similarities between apoptotic cell death and CTL or NK cell mediated cytolysis, which suggest that these cells activate an endogenous cell death programme in the target cell. From available data it is supposed that CTL-mediated cytolysis involves two mechanisms: (1) engagement of the CTL–TCR with an antigen/receptor on the surface of the target cell results in activation of the cell death programme in the target cell; and (2) the same engagement stimulates the release from the CTL of lytic granules that contain perforins and fragmentins, which damage the cellular membrane of the target cell. The relative contributions of membrane and apoptotic damage in CTL mediated cytolysis are unknown. NK cells, together with TNF, secreted by TH cells/macrophages, are able to trigger apoptosis in many cell types. When T cells are deprived of cytokines, then mature T cells undergo apoptosis. T cells also undergo apoptosis, when the triggering of the auxillary surface molecule CD4 of T cells occurs, uncoupled from TCR triggering.[77] For instance, by this mechanism, CD4 triggering of T lymphocytes by the HIV-1 viral envelope gp-120 may induce apoptosis of T lymphocytes that are not even infected, thus explaining the T-cell depletion in AIDS patients.[78]

*Genes:* Several genes have been associated with apoptosis, either because they are expressed or not during apoptosis and because their expression/suppression affects the process of apoptosis. A very characteristic biochemical event in apoptosis is the double strand cleavage of nuclear DNA at the linker regions between the nucleosomes. This results in a number of 180–200 base pair fragments and multiples of it, which become visible as a ladder pattern on agarose gel electrophoresis of the DNA extracted from apoptotic cells. In contrast, in necrosis the DNA breakdown occurs randomly, which results in DNA fragments that present as a smear in electrophoresis of DNA extracted from necrotic cells. DNA damage induced by radiation or cytotoxic drugs can lead to apoptosis. It is even likely that most chemotherapeutic drugs induce tumour cell apoptosis rather than inhibit tumour cell proliferation.[37] It has been shown that there is a lag period of several hours between the triggering and the occurrence of apoptosis, suggesting the probable need for RNA and protein synthesis. Administration of RNA and protein synthesis-inhibitors results in suppression or delay or apoptosis. Knowledge about the genes that regulate the intracellular biochemical processes is still fragmentary. A number of genes and gene products* have been implicated in the control of apoptosis. In mammalian systems these include: *Fas*/APO=1, *bcl-2, (c-myc c-myb, c-fos, c-jun)*, p53, *ced-3*/ICE, TRPM-2/SGP. In the following sections details are given of those genes and gene products where a role in the apoptotic process has been demonstrated.

Fas *gene/APO-1 molecule. Fas* is a gene, whose product (Fas) is a membrane-spanning protein, homologous to tumour necrosis factor (TNF) receptor and nerve growth factor (NGF) receptor.[79,80] TNF induces surface receptor apoptosis in target cells via this cell. The *Fas* product is identical to the cell surface molecule APO-1.[81] *Fas*/APO-1 cross-linking by antibody induces apoptosis, which indicates that *Fas* product, APO-1, mediates an apoptotic signal into the cells. Apo-1 has been shown to be expressed in human B lymphocytic leukaemia cells. *Bcl-2* expression is down-regulated and APO-1 expression upregulated by exposition to IL-2, which prepares the cells for anti-*Fas*/Apo-1 mediated apoptosis.[82] The *Fas*/APO-1 system has also been shown to be the target molecule for cytotoxic T cells.[83] Mice bearing the *lpr* mutation are defective in *Fas*, and the thymoma, that they develop is not due to T cell proliferation but to cell accumulation, because the thymocytes die less readily than their normal counterparts.[84]

Bcl-2 *gene.* The *bcl-2* oncogene was first identified as a gene over-expressed in human follicular B-cell lymphomas, following reciprocal chromosomal translocation t(14; 18)(q32; q21). In this aberration a translocation of the proto-oncogene *bcl-2* (B Cell Lymphoma-2) on chromosome 18 to the IgH locus on the JH segment of the immune gene on chromosome 14 has occurred. When over-expressed *bcl-2* inhibits apoptosis in a variety of cells including cytokine/growth factor-deprived haematopoietic cells and neurons.[85–87] *Bcl-2* does not stimulate cell proliferation, but promotes survival of cells in a non-cycling state. *Bcl-2* co-operates with disregulated *c-myc* expression to promote haematopoietic tumour development in transgenic mice. Since *bcl-2* inhibits induction of apoptosis by *c-myc*, the basis of this co-operation is presumably the preservation from apoptosis by *bcl-2* of *c-myc*-expressing target cells, which then can proliferate. *Bcl-2* renders cells less sensitive to radiation and to cytotoxic drugs; only CTL killing is not inhibited.[86] The mechanism by

* In the text, genes are given in italic characters and gene products in roman characters.

which *bcl-2* blocks apoptosis is unknown, but it may be related to the fact that the gene product resides at the inner side of the mitochondria, which remain intact during a large part of the apoptotic process. An attractive hypothesis is that *bcl-2* protects cells by inhibiting lipid peroxidation, even in the presence of reactive oxygen intermediates.[88] Recently, a number of *bcl-2*-related genes have been identified, including *bax, bcl-x, mcl1, E1B* (in adenovirus), *LMW5-HL* (in African swine fever virus), *BHRF1* (in Epstein–Barr virus).

C-myc *gene.* Growth factors induce expression of the oncogene *c-myc*, which indicates that expression of *c-myc* is associated with cell proliferation.[89] Otherwise, deprivation of growth factors causes down-regulation of *c-myc* and is accompanied by growth arrest or induces in cells with a high expression of *c-myc* apoptosis.[90] The *c-myc* protein has apparently a dual role: (1) promoting proliferation in the presence of a relative abundance of appropriate growth factors; and (2) inducing apoptosis when *c-myc* is expressed in cells during growth arrest.[91] Vaux *et al.*[85] showed that expression of the *bcl-2* gene in pre-B cells of transgenic mice with a high expression of *c-myc* promotes survival of these cells, which means that *bcl-2* co-operates with *c-myc* to immortalize pre-B cells. The role of growth factor in relation to *c-myc* expression can be summarized as follows :

Growth factor (GF)

→ *c-myc* expression ↑ → cell proliferation

GF deprivation

→ *c-myc* expression ↓ → cell in GO

GF deprivation

+ *c-myc* expression ↑↑ → apoptosis

*p53 protein.* Many studies concerning the p53 protein have shown that this protein has a tumour suppressor activity, which is illustrated by the fact that p53 is the most commonly disrupted gene in human malignancies.[92] It has been demonstrated that the p53 protein forms a complex with the cell cycle regulating protein cdc2-kinase after which the cell becomes arrested in the G1-phase or may switch to a differentiation mode.[93] In cells where DNA damage has occurred, e.g. after UV or gamma irradiation or after chemotherapy, the p53 protein accumulates and cell proliferation is arrested.[94–98] Possibly this mechanism is important for preventing reproduction of DNA damage and to give the cell time for DNA repair. This capacity of p53 may have the function of eliminating those cells in which DNA repair has not been successful. Cells that lack active p53 protein, because of mutation or binding onto other cellular or viral proteins, are not arrested in the G1-phase and do not undergo apoptosis.[99] Such cells are consequently genetically less stable and may develop malignant cell clones.[100] Circumstantial evidence exists to postulate that p53 induces survival factor dependence in the involved cell.[101]

The role of the p53 protein can be summarized as follows:

DNA damage → p53 protein ↑
→ increases survival factor dependence
→ arrest in G1 until repair is completed
→ apoptosis if repair fails
DNA damage → p53 protein ↓
→ mitotic failure, cell death
→ progress to malignancy ced-3/*ICE.* Recently a cell suicide gene responsible for programmed cell death in *Caenorhabditis elegans* (*ced-3*) has been cloned.[102] It was demonstrated that the gene product is a protein, very similar to interleukin-1-converting enzyme (ICE). ICE, a cysteine protease, cleaves the inactive precursor of IL-1 to generate an active cytokine. ICE causes apoptosis in rat fibroblasts in which the ICE gene was introduced and brought to expression.[103] In addition it was found that *bcl-2* could inhibit the cell death brought on by over-expression of ICE gene. The researchers concluded that members of the *ced-3*/ ICE gene family might function in apoptotic cell death in vertebrates.

MTS1. *MTS1* (multiple tumor suppressor 1) encodes for a previously identified inhibitor (p16) of cyclin-dependent kinase 4 (cdk-4). This gene product prevents the activation of *c-myc* by blocking an enzyme in the pathway that allows messages from the cell surface receptors to switch on *c-myc*. In 46% of 290 different human cancers functional copies of *MTS1* are missing leaving *c-myc* unchecked and the pathway to cell division open.[104]

### Apoptosis and Inflammation

*Bacterial infections:* The principal event that leads to inflammatory disease is cell damage, which can be induced by bacterial or viral toxins, chemical or physical injury, anoxia, starvation, or allergic auto-immune attack. Massive cell damage and necrosis result in leakage of cell contents into the adjacent tissues. Dissipation of enzymes and mediators of inflammation, released from the necrotic cells, results in expression of adhesive molecules on endothelial cells and on the neutrophils. Receptors on granulocytes and monocytes (and lymphocytes), such as Mac-1, LFA-1 and P150.95 (VLA-4), with high affinity for the adhesion peptides (integrins), interact with their counterparts on the endothelium (ELAM-1, ICAM-1, ICAM-2 and VCAM-1). This mechanism promotes the capillary transmigration of these cells to the injured tissue. The expression of adhesins is upregulated by IL-1, TNF and by exposure to chemotaxins such as C′5a, IL-8. The accumulation of neutrophils and release of enzymes and oxygen radicals from the attracted granulocytes underlie the inflammatory reaction.

An inflammatory reaction is not always a salutory response to injurious events. Neutrophil granulocytes have been implicated in the pathogenesis of a wide variety of diseases.[105] They contain a large number of agents with the capacity to injure tissue and to degrade matrix proteins into chemotactic fragments.[106] Persistent accumulation of inflammatory cells is associated with destruction of tissue matrix or deposition of scar tissue and can lead to the loss of organ function. Examples of such catastrophic organ damage are given by lung emphysema, respiratory distress syndrome, fibrosing alveolitis, pneumoconiosis, liver cirrhosis, forms of glomerulonephritis, and rheumatoid arthritis.

For inflammatory tissues to return to normal, all the events involved in the evolution of inflammation must be reversed. These must include removal of the inciting stimulus, cessation of neutrophil accumulation, cessation of further release of histotoxic and pro-inflammatory mediators, return of microvascular permeability to normal and cessation of monocyte emigration from blood vessels. The tissue monocytes must transmutate into macrophages, which remove extravasated fluid, proteins, bacterial and cellular debris and accumulated neutrophils. Cessation of neutrophil and monocyte emigration may occur after dissipation of chemotactic factors from the inflamed tissue, but the mechanism for the loss of chemotaxins has not yet been identified.

Recent reviews dealing with the general aspects of inflammation have been given by Saukkonen *et al.*,[107] Haslett,[108] Dinarello and Wolff,[109] and Bonta and Ben-Efraim.[110] The following paragraph is restricted to the role of apoptosis in the clearance of granulocytes from the 'battlefield' of inflammation by macrophages.

Granulocytes, where they have infiltrated, release enzymes, oxygen radicals, cytokines and mediators of inflammation, locally. All these products may trigger potentially dangerous responses within the organism. This aspect of inflammation has led to much research into enzyme inhibitors, oxygen radical scavengers and membrane stabilizers, but little attention has been paid to factors controlling the accumulation and the tissue load of granulocytes and their histotoxic products.

Tissue kinetics of neutrophils depend upon the half-life of the neutrophils, the amount of neutrophils that enter the tissue per time unit, and the speed by which they are removed. Lee *et al.*[111] have shown that bacterial products such as lipopolysaccharide, the chemotactic peptides C5a and GM-CSF, all exert an inhibitory effect on the rate of neutrophil apoptosis. Concentrations of these mediators are likely to be high in acutely infected tissues, but vary considerably during resolution and in chronic inflammation. Neutrophil apoptosis may represent an important event in the control of inflammation, eliminating the neutrophil for disposal and for its capacity to generate and release histotoxic products.[47] The efficient removal of neutrophils from inflamed sites, once their biological purpose (killing of invading pathogens, breakdown of cell debris) is completed, is a highly desirable and effective mechanism on the road to reconstitution of the normal situation. Neutrophils can undergo apoptosis and the associated changes of the cell surface aid their phagocytosis by macrophages.[46] It has been assumed that granulocytes disintegrate to apoptotic bodies before their fragments are removed by local macrophages.[112] Removal of neutrophils from the inflammatory site, without release of granule contents, is of paramount importance for cessation of inflammation. Neutrophil apoptosis is accelerated by TNFα.[113] This process operates wherever a need exists to curtail a suppurative exudation, be it the resolution of an exsudative infection or the obturation of an abscess cavity.

The rate of apoptosis of human eosinophils, already slower than that reported for neutrophils, is delayed *in vitro* by the eosinophil differentiation factor IL-5.[24,58,59]

The processes involved in the control of monocyte recruitment have not yet been elucidated, although very likely they are governed by similar principles as described under neutrophil emigration. Macrophages play an important role in reconstitution of normal haemodynamics and recovery of tissue integrity after inflammation. Monocytes are mobilized to the site of inflammation by C'5a, MLP, MCP-1 and TGFβ. Their elimination from the inflammatory site presumably takes place mostly by apoptosis, which is promoted by IL-4 (←TH-2 cells) and inhibited by GM-CSF/M-CSF, IL-3, IFNγ(←TH-1 cells), IL-1 and TNFα.[114]

*Viral infections:* Viruses have developed strategies to inhibit apoptosis following virus infection, which serves to prolong the life of their target cells and promote virus replication. Epstein–Barr virus induces the expression of *bcl-2* related genes in the infected cells and encodes for a *bcl-2* homologue,[115] including E1B in adenovirus, LMW5-HL in African swine fever virus, BHRF1 in Epstein–Barr virus infection.[115–118] Presumably, adenovirus, human papilloma viruses and SV40 modulate apoptosis also by interaction of viral proteins with cellular P53.

It has recently been suggested that both qualitative and quantitative defects in CD4+ T cells in patients with HIV infection may be the result of activation-induced cell death by apoptosis.[119–122] Since apoptosis can be induced in mature murine CD4+ T cells after cross-linking of CD4 molecules, uncoupled from TCR triggering, there has been speculation that crosslinking of the CD4 molecule by HIV gp120 or by gp120/anti-gp120 immune complexes prepares the cell for apoptosis that occurs when MHC class II molecule presents an antigen to the TCR.[77,121,122] Thus, the mere activation of a prepared cell by a specific antigen or superantigen could lead to the death of the cell, without direct infection by HIV. Apoptosis of CD8+ cells has been obscured *in vitro* even in the absence of an antigenic stimulus.[123,124]

### Concluding remarks

Cell death plays an important role in immunogenesis, during inflammation and in the resolution of inflammatory reactions. The mechanisms and mediators involved have not yet been fully elucidated, but there is evidence that surrounding cells adjacent to inflammation kill themselves by apoptosis, increasing the damage caused by inflam-

TISSUE INJURY

bacterial products, toxins, immune complexes, physical injury, cytokines

↓

Chemotaxis

| | |
|---|---|
| PG 1-2. PGD/E/F | vasodilatation |
| 5-HETE | chemotaxis |
| PAF (←endothelial cells) | chemotaxis, increased permeability, leukocyte aggregation |
| IL-8(←monocytes) | attractant of granulocytes |
| Leukotrienes | increased permeability/oedema |
| FXII activation | kinin formation, coagulation, fibrinolysis |
| Complement activation | C'5a → chemotaxis, C'3b → phagocytosis |

↓

Cell adhesion

| | |
|---|---|
| C'5a, TNF | → surface expression on leukocytes of integrins: LFA-1, Mac-1, P150-95 |
| IL-1, TNF | → surface expression on endothelial cells adhesins: ELAM-1, ICAM-1, VCAM-1 |

↓

Leukocyte margination, Rolling, Adhesion, Emigration, Accumulation

↓

INFLAMMATORY REACTION

| | |
|---|---|
| Granulocytes | release lysosomal enzymes |
| | oxygen free radicals |
| | nitric oxide |
| Monocytes | mobilization, activation, transformation by cytokines |
| | (C'5a,FMLP,IF,IL-1,MCP-1,TGF-β,TNF) |
| | transformation→ |
| Macrophages | release proteases, collagenases |
| | nitric oxide |
| | arachidonic acid metabolites |
| | fibrinogenic cytokines |
| | angiogenesis (FGF) |
| | growth factors (CSFs,PDGF,FGF,TGF-β) |
| | cytokines (IL-1,IL-8,TNF) |
| | transformation→ |
| Epitheloid cells | induced by IF-γ (←T-cell mediated immunity) |

↓

APOPTOSIS of surrounding tissue cells

APOPTOSIS of granulocytes, enhanced by TNFα, suppressed by CSF, C'5a

APOPTOSIS of monocytes, enhanced by IL-4, suppressed by IL-1β,CSF,IFN-γ,TNFα

↓

RESOLUTION INFLAMMATION

FIG. 1. The role of granulocyte recruitment or accumulation and granulocyte/monocyte apoptosis in the evolution and resolution of inflammation. Abbreviations: C'3b/C'5a, complement factors; CSFs, colony stimulating factors; FXII, Hageman factor; FGF, fibroblast growth factor; FLMP, formyl-methionyl-leucyl-phenylalanine; HETEs, hydroperoxy derivatives of arachidonic acid; IFN, interferon; ILs, interleukins; MCP-1, monocyte chemotactic protein-1; PAF, platelet-activating factor; PDGF, platelet-derived growth factor; PG1, 2, D, E, F, prostaglandins; TGF-β, transforming growth factor-β; TNF, tumour necrosis factor.

matory reactions. In Fig. 1 the various biological processes that mediate and modulate the inflammatory reaction and the role of apoptosis are depicted schematically. The facts known so far have been put together in this review in order to draw attention to apoptosis and to stimulate further research on its role in inflammatory diseases.

### References

1. Glücksmann A. Cell deaths in normal vertebrate ontogeny. *Biol Rev Philos Soc* 1951; **26**: 59–86.
2. Saunders JW. Death in embryonic systems. *Systems* 1966; **154**: 604–612.
3. Wyllie AH, Kerr JFR, Currie AR. Cell death: the significance of apoptosis. *Int Rev Cytol* 1980; **68**: 251–300.
4. Umansky SR. The genetic program of cell death. Hypothesis and some applications: transformation, carcinogenesis, ageing. *J Theor Biol* 1982; **97**: 591–602.
5. Trump BF, Berezesky IK, Cowley RA. The cellular and subcellular characteristics of acute and chronic injury with emphasis on the role of calcium. In: Cowley RA, Trump BF (eds). *Pathophysiology of Shock, Anoxia and Ischemia*. Baltimore, MD: Williams & Wilkins, 1982: 6–46.
6. Kerr JFR, Wyllie AH, Currie AR. Apoptosis: a basic biological phenomenon with wide-ranging implications in tissue kinetics. *Br J Cancer* 1972; **26**: 239–257.
7. Ellis RE, Yuan J, Horvitz HR. Mechanisms and functions of cell death. *Annu Rev Cell Biol* 1991; **7**: 663–698.
8. Alison MR, Sarraf CE. Apoptosis: a gene-directed programme of cell death. *J R Coll Physicians, London* 1992; **26**: 25–35.
9. Cohen JJ. Apoptosis. *Immunol Today* 1993; **14**: 126–130.
10. Schwartzman RA, Cidlowski JA. Apoptosis: the biochemistry and molecular biology of programmed cell death. *Endocr Rev* 1993; **14**: 133–151.
11. Gewitz A. DNA damage, gene expression, growth arrest and cell death. *Oncol Res* 1994; **5**: 397–417.
12. Vermes I, Haanen C. Apoptosis and programmed cell death in health and disease. *Adv Clin Chem* 1994; **31**: 178–246.
13. Sandow BA, West NB, Normal RL, Brenner RM. Hormonal control of apoptosis in hamster uterine luminal epithelium. *Am J Anat* 1979; **156**: 15–35.
14. Rotello RJ, Lieberman RC, Lepoff RB, Gerschenson LE. Characterization of uterine epithelium apoptotic cell death kinetics and regulation by progesterone and RU486. *Am J Path* 1991; **140**: 449–456.
15. Kyprianou N, English HF, Davidson NE, Isaacs JT. Programmed cell death during regression of the MCF-7 human breast cancer following estrogen ablation. *Cancer Res* 1991; **51**: 162–166.
16. Wielckens K, Delfs T. Glucocorticoid-induced cell death poly-[adenosine diphosphate (ADP)–ribosyl]-ation: increased toxicity of dexamethasone on mouse S49.1 lymphoma cells with the poly(ADP-ribosylation) inhibitor benzamide. *Endocrinology* 1986; **119**: 2383–2392.
17. Dieken ES, Miesfeld RL. Transcriptional transactivation functions localized to the glucocorticoid receptor N terminus are necessary for steroid induction of lymphocyte apoptosis. *Mol Cell Biol* 1992; **12**: 589–597.
18. Raff MC. Social control on cell survival and cell death. *Nature* 1992; **356**: 397–400.
19. Hefti F, Knusel B. Chronic administration of nerve growth factor and other neurotrophic factors to the brain. *Neurobiol Aging* 1988; **9**: 689–690.
20. Edwards SN, Buckmaster AE, Tolkovsky AM. The death programme in cultured sympathetic neurons can be suppressed at the post-translational level by nerve growth factor, cyclic AMP and depolarization. *J Neurochem* 1991; **57**: 2140–2143.
21. Svrzic D, Schubert D. Insulin-like growth factor 1 supports embryonic nerve cell survival. *Biochem Biophys Res Commun* 1990; **172**: 54–60.
22. Drago J, Murphy M, Carroll SM, Harvey RP, Brtlett PF. Fibroblast growth factor-mediated proliferation of central nervous system precursors depends on endogenous production of insulin-like growth factor I. *Proc Natl Acad Sci USA* 1991; **88**: 2199–2303.
23. Barres BA, Hart IK, Coles HSR, Burne JF, Voyvodic JT, Richardson WD, Raff MC. Cell death and control of cell survival in the oligodendrocyte lineage. *Cell* 1992; **70**: 31–46.
24. Stern M, Meagher L, Savill J, Haslett C. Apoptosis in human eosinophils. Programmed cell death in the eosinophil leads to phagocytosis by macrophages and is modulated by IL-5. *J Immunol* 1992; **148**: 3543–3549.
25. Louis J-C, Magal E, Takayama S, Varon S. CNTF protection of oligodendrocytes against natural and tumor necrosis factor-induced death. *Science* 1993; **259**: 689–692.
26. Harrington EA, Bennett MR, Fanidi A, Evan GI. c-Myc-induced apoptosis in fibroblasts is inhibited by specific cytokines. *EMBO J* 1994; **13**: 3286–3295.
27. Williams GT, Smith CA, Spooncer E, Dexter TM, Taylor DR. Haemopoietic colony stimulating factors promote cell survival by suppressing apoptosis. *Nature* 1990; **343**: 76–79.
28. Rodriguez-Tarduchy G, Collins MKL, García I, López-Rivas A. Insulin-like growth factor-1 inhibits apoptosis in IL-3-dependent haemopoietic cells. *J Immunol* 1992; **149**: 535–540.
29. Koury MJ, Bondurant MC. Erythropoietin retards DNA breakdown and prevents programmed death in erythroid progenitor cells. *Science* 1990; **248**: 378–381.
30. Spivak JL, Pham T, Isaacs M, Hankins WP. Erythropoietin is both a mitogen and a survival factor. *Blood* 1991; **77**: 1228–1233.
31. Metcalf D, Merchav S. Effects of GM-CSF deprivation on precursors of granulocytes and macrophages. *J Cell Physiol* 1982; **112**: 411–413.
32. Yamamoto C, Yoshida S-I, Taniguchi H, Qin MH, Miyamoto H, Mizuguchi Y. Lipopolysaccharide and granulocyte colony-stimulating factor delay neutrophil apoptosis and ingestion by guinea pig macrophages. *Infection Immunity* 1993; **61**: 1972–1979.
33. Kannan Y, Usami K, Okada M, Shimizu S, Matsuda H. Nerve growth factor suppresses apoptosis of murine neutrophils. *Biochem Biophys Res Commun* 1992; **186**: 1050–1056.
34. Araki S, Shimada Y, Kaji K, Hayashi Y. Apoptosis of vascular endothelial cells by fibroblast growth factor deprivatiion. *Biochem Biophys Res Comm* 1990; **168**: 1194–1200.
35. Kessler JA, Ludlam WH, Freidin MM, *et al.* Cytokine-induced programmed death of cultured sympathetic neurons. *Neuron* 1993; **11**: 1123–1132.
36. Takeda Y, Watanabe H, Yonehara S, Yamashita T, Saito S, Sendo F. Rapid acceleration of neutrophil apoptosis by tumor necrosis factor-a. *Intern Immunol* 1993; **5**: 691–694.
37. Collins MKL, Marvel J, Malde P, Lopez-Rivaz A. IL-3 protects bone marrow cells from apoptosis induced by DNA damaging agents. *J Exp Med* 1992; **176**: 1043–1051.
38. Ormerod MG, Collins MKL, Rodriguez-Tarduchy G, Robertson D. Apoptosis in interleukin-3-dependent haemopoietic cells. *J Immunol Meth* 1992; **153**: 57–65.
39. Tushinski RJ, Oliver IT, Guilbert LJ, Tynan PW, Stanley ER. Survival of mononuclear phagocytes depends on a lineage-specific growth factor that the differentiated cells selectively destroy. *Cell* 1982; **28**: 71–81.
40. Begley CG, Lopez AF, Nicola NA, Warren DJ, Vadas MA, Sanderson CJ, Metcalf D. Purified colony-stimulating factors enhance the survival of human neutrophils and eosinophils *in vitro*: a rapid and sensitive microassay for colony-stimulating factors. *Blood* 1986; **68**: 162–166.
41. Rothenberg ME, Owen WF, Silberstein DS, Woods J, Soberman RJ, Austen KF, Stevens RL. Human eosinophils have prolonged survival, enhanced functional properties, and become hypodense when exposed to human interleukin 3. *J Clin Invest* 1988; **81**: 1986–1992.
42. Rodriguez-Tarduchy G, Collins M, Lopez-Rivas A. Regulation of apoptosis in interleukin-3-dependent haemopoietic cells by interleukin-3 and calcium ionophores. *EMBO J* 1990; **9**: 2997–3002.
43. Collins MKL, Marvel J, Malde P, Lopez-Rivaz A. IL-3 protects bone marrow cells from apoptosis induced by DNA damaging agents. *J Exp Med* 1992; **176**: 1043–1051.
44. Koury MJ, Bondurant MC. Erythropoietin retards DNA breakdown and prevents programmed death in erythroid progenitor cells. *Science* 1990; **248**: 378–380.
45. Yu H, Bauer B, Lipke GK, Phillips RL, Van Zant G. Apoptosis and hematopoiesis in murine fetal liver. *Blood* 1993; **81**: 373–384.
46. Savill JS, Wyllie AH, Henson JE, Walport MJ, Henson PM, Haslett C. Macrophage phagocytosis of ageing neutrophils in inflammation: Programmed cell death in the neutrophil leads to its recognition by macrophages. *J Clin Invest* 1989; **83**: 865–875.
47. Whyte MKB, Meagher LC, MacDermot J, Haslett C. Impairment of function in aging neutrophils is associated with apoptosis. *J Immunol* 1993; **150**: 5124–5134.
48. Kizaki H, Nakada S-i, Ohnishi Y, Azuma Y, Mizuno Y, Tadakuma T. Tumour necrosis factor-a enhances cAMP-induced programmed cell death in mouse thymocytes. *Cytokine* 1993; **5**: 342–347.
49. Hernández-Caselles T, Stutman O. Immune functions of tumor necrosis factor. I. Tumor necrosis factor induces apoptosis of mouse thymocytes and can also stimulate or inhibit IL-6-induced proliferation depending on the concentration of mitogenic costimulation. *J Immunol* 1993; **151**: 3999–4012.
50. Wright SC, Kumar P, Tam AW, Shen N, Varma M, Larrick JW. Apoptosis and DNA fragmentation precede TNF-induced cytolysis in U937 cells. *J Cell Biochem* 1992; **48**: 344–355.
51. Lotem J, Sachs L. Haematopoietic cytokines inhibit apoptosis induced by transforming growth factor b1 and cancer chemotherapy compounds in myeloid leukemic cells. *Blood* 1992; **80**: 1750–1757.
52. Bishop CJ, Moss DJ, Ryan JM, Burrows SR. T lymphocytes in infectious mononucleosis. II. Response *in vitro* to interleukin-2 and establishment of T cell lines. *Clin Exp Immunol* 1985; **60**: 70–77.
53. Duke RC, Cohen JJ. IL-2 addiction: withdrawal of growth factor activates a suicide programme in dependent T cell. *Lymphokine Res* 1986; **5**: 289–299.
54. Lenardo MJ. Interleukin-2 programs mouse ab T lymphocytes for apoptosis. *Nature* 1991; **353**: 858–861.
55. Migliorati G, Nicoletti I, Pagliacci MC, D'Adamio L, Riccardi C. Interleukin-2 induces apoptosis in mouse thymocytes. *Cell Immunol* 1993; **146**: 52–61.
56. Zubiaga AM, Munoz E, Huber BT. IL-4 and IL-2 selectivity rescue Th cell subsets from glucocorticoid-induced apoptosis. *J Immunol* 1992; **149**: 107–112.
57. Panayiotidis P, Ganeshaguru K, Jabbar SAB, Hoffbrand AV. Interleukin-4 inhibits apoptotic cell death and loss of the bcl-2 protein in B-chronic lymphocytic leukaemia cells *in vitro*. *Br J Haematol* 1993; **85**: 439–445.
58. Yamaguchi Y, Suda T, Ohta S, Tominaga K, Miura Y, Kasahara T. Analysis of the survival of mature human eosinophils: interleukin-5 prevents apoptosis in mature human eosinophils. *Blood*; **78**: 2542–2547.
59. Her E, Frazer EJ, Austen KF, Owen WF. Eosinophil hematopoietins antagonize the programmed cell death of eosinophils. Cytokine and glucocorticoid effects on eosinophils maintained by endothelial cell-conditioned medium. *J Clin Invest* 1991; **88**: 1982–1987.
60. Hogquist K, Nett MA, Unanue ER, Chaplin DD. Interleukin 1 is processed and released during apoptosis. *Proc Natl Acad Sci USA* 1991; **88**: 8485–8489.
61. Mangan DF, Welch GR, Wahl SM. Lipopolysaccharide, tumor necrosis factor-a, and IL-1b prevent programmed cell death (apoptosis) in human peripheral blood monocytes. *J Immunol* 1991; **146**: 1541–1546.
62. Mangan DF, Robertson B, Wahl SM. IL-4 enhances programmed cell death (apoptosis) in stimulated human monocytes. *J Immunol* 1992; **148**: 1812–1816.
63. Mekori YA, Oh CK, Metcalfe DD. IL-3 dependent murine mast cells undergo apoptosis on removal of IL-3. *J Immunol* 1993; **151**: 3775–3784.
64. Kobayashi S, Teramura M, Sugawara I, Oshimi K, Mizoguchi H. Interleukin-11 acts as an autocrine growth factor for human megakaryoblastic cell lines. *Blood* 1993; **81**: 889–893.
65. Kappler JW, Roehm N, Marrack P. T cell tolerance by clonal elimination in the thymus. *Cell* 1987; **49**: 273–280.
66. MacDonald HR, Lees RK. Programmed death of autoreactive thymocytes. *Nature* 1988; **343**: 642–644.
67. Smith CA, Williams GT, Kingston R, Jenkinson EJ, Owen JJT. Antibodies to CD3/T-cell receptor complex induce death by apoptosis in immature T cells in thymic cultures. *Nature* 1989; **337**: 181–184.
68. Von Boehmer H. Thymic selection: a matter of life and death. *Immunol Today* 1992; **13**: 454–458.
69. Liu YJ, Mason DY, Johnson GD. Germinal center cells express bcl-2 protein after activation by signals which prevent their entry into apoptosis. *Eur J Immunol* 1991; **21**: 1905–1910.
70. Kroemer G, Martínez-A C. Mechanisms of self tolerance. *Immunol Today* 1992; **13**: 401–404.
71. Liu YJ, Joshua DE, Williams GT, Smith CA, Gordon J, MacLennan ICM. Mechanism of antigen-driven selection in germinal centres. *Nature* 1989; **342**: 929–931.
72. Golstein P, Ojcius DM, Young JD-E. Cell death mechanisms and the immune system. *Immunol Rev* 1991; **121**: 29–65.
73. Von Boehmer H. Developmental biology of T-cells in T-cell receptor transgenic mice. *Ann Rev Immunol* 1990; **9**: 531–536.
74. Cohen JJ, Duke RC. Apoptosis and programmed cell death in immunity. *Ann Rev Immunol* 1992; **10**: 267–293.
75. Fesus L. Apoptosis. *Immunol Today* 1992; **13**: A16–A17.
76. Kabelitz D, Pohl T, Pechhold K. Activation-induced cell death (apoptosis) of mature peripheral lymphocytes. *Immunol Today* 1993; **14**: 338–339.
77. Newell MK, Haughn LI, Maroun CR, Julius MH. Death of mature T cells by separate ligation of CD4 and the T-cell receptor for antigen. *Nature* 1990; **347**: 286–289.
78. Ameisen JC. Programmed cell death and AIDS: from hypothesis to experiment. *Immunol Today* 1992; **13**: 3388–3391.
79. Itoh N, Yonehara S, Ishii A, *et al.* The polypeptide encoded by the cDNA for human cell surface antigen Fas can mediate apoptosis. *Cell* 1991; **66**: 233–243.
80. Itoh N, Nagata S. A novel protein domain required for apoptosis. *J Biol Chem* 1993; **268**: 10932–10937.
81. Oehm A, Behrmann I, Falk W, *et al.* Purification and molecular cloning of the APO-1 cell surface antigen, a member of the tumor necrosis factor/nerve growth factor receptor superfamily. Sequence identity with the Fas antigen. *J Biol Chem* 1992; **267**: 10709–10715.
82. Mapara MY, Bargou R, Zugek C, *et al.* APO-1 mediated apoptosis or proliferation in human chronic B lymphocytic leukemia: correlation with bcl-2 oncogene expression. *Eur J Immunol* 1993; **23**: 702–708.
83. Rouvier E, Luciani MF, Golstein P. Fas involvement in $Ca^{2+}$ independent T cell mediated cytotoxicity. *J Exp Med* 1993; **177**: 195–200.
84. Watanabe-Fukunaga R, Brannan CI, Copeland NG, Jenkins NA, Nagata S. Lymphoproliferation disorder in mice explained by defects in Fas antigen that mediates apoptosis. *Nature* 1992; **356**; 314–317.
85. Vaux DL, Cory S, Adams JM. Bcl-2 gene promotes haemopoietic cell survival and cooperates with c-myc to immortalize pre-B cells. *Nature* 1988; **335**: 440–442.
86. Vaux DL, Aguila HL, Weissman IL. Bcl-2 prevents death of factor-deprived cells but fails to prevent apoptosis in targets of cell mediated killing. *Int Immunol* 1992; **4**: 821–824.
87. Nunez G, London L, Hockenberry DM, Alexander M, McKearn JP, Korsmeyer SJ. Deregulated Bcl-2 gene expression selectively prolongs survival of growth factor-deprived hemopoietic cell lines. *J Immunol* 1990; **144**: 3602–3610.
88. Wyllie A. Death gets a brake. *Nature* 1994; **369**: 272–273.
89. Waters C, Littlewood T, Hancock D, Moore J, Evan G. C-myc protein expression in untransformed fibroblasts. *Oncogene* 1991; **6**: 101–109.
90. Evan GI, Wyllie AH, Gilbert CS, *et al.* Induction of apoptosis in fibroblasts by c-myc protein. *Cell* 1992; **69**: 119–128.
91. Evan GI, Littlewood TD. The role of c-myc in cell growth. *Curr Opin Gene Dev* 1993; **3**: 44–49.
92. Hollstein M, Sidransky D, Vogelstein B, Harris C. P53 mutations in human cancers. *Science* 1991; **253**: 49–53.
93. Sturzbecher H, Maimets T, Chumakov P, *et al.* P53 interacts with p34 cdc-2 in mammalian cells: implications for cell cycle control and oncogenesis. *Oncogene* 1990; **5**: 795–801.
94. Kastan MB, Onyekwere O, Sidransky D, Vogelstein B, Craig RW. Participation of p53 protein in the cellular response to DNA damage. *Cancer Res* 1991; **51**: 6304–6311.
95. Lane DP. P53, guardian of the genome. *Nature* 1992; **358**: 15–16.
96. Maltzman W, Czyzyk L. UV irradiation stimulates levels of p53 cellular tumor antigen in nontransformed mouse cells. *Mol Cell Biol* 1984; **4**: 1689–1694.
97. Shaw P, Bovey R, Tardy S, Sahli R, Sordat B, Costa J. Induction of apoptosis by wild-type p53 in a human colon tumor derived cell line. *Proc Natl Acad Sci USA* 1992; **89**: 4495–4499.
98. Yonisch-Rouach E, Resnitzky D, Lotem J, Sachs L, Kimchi A, Oren M. Wild-type p53 induces apoptosis of myeloid leukaemic cells that is inhibited by interleukin-6. *Nature* 1991; **352**: 345–347.
99. Oliner JD, Kinzler KW, Meltzer PS, George DL, Vogelstein B. Amplification of a gene encoding a p53-associated protein in human sarcomas. *Nature* 1992; **358**: 80–83.
100. Perry ME, Levine AJ. Tumor-suppressor p53 and the cell cycle. *Curr Opin Genet Dev* 1993; **3**: 50–54.
101. Gottlieb E, Haffner R, von Rüden T, Wagner EF, Oren M. Down-regulation of wild-type p53 activity interferes with apoptosis of IL-3-dependent hematopoetic cells following IL-3 withdrawal. *EMBO J* 1994; **13**: 1368–1374.
102. Yuan J, Shaham S, Ledoux S, Ellis HM, Horvitz HR. The C elegans cell death gene ced-3 encodes a protein similar to mammalian interleukin-1-converting enzyme. *Cell* 1993; **75**: 1–20.
103. Miura M, Zhu H, Rotello R, Hartwieg EA, Yuan J. Induction of apoptosis in fibroblasts by IL-1-converting enzyme, a mammalian homolog of the C elegans cell death gene ced-3. *Cell* 1993; **75**: 653–660.
104. Kamb A, Gruis NA, Weaver-Feldhaus J, *et al. Science* 1994; **264**: 436–440.
105. Weiss SJ. Tissue destruction by neutrophils. *N Engl J Med* 1989; **320**: 365–376.
106. Vartio T. Seppa H, Vaheri A. Susceptibility of soluble and matrix fibronectin to degradation by tissue proteinases, mast cell chymase and cathepsin. *J Biol Chem* 1981; **256**: 471–477.
107. Saukkonen K, Sande S, Cioffe C, Wolpe S, Sherry B, Cerami A. The role of cytokines in the generation of inflammation and tissue damage in experimental Gram-positive meningitis. *J Exp Med* 1990; **171**: 439–448.
108. Haslett C. Resolution of acute inflammation and the role of apoptosis in the tissue fate of granulocytes. *Clin Sci* 1992; **83**: 639–648.
109. Dinarello CA, Wolff SM. The role of interleukin-1 in disease. *New Engl J Med* 1993; **328**: 106–113.
110. Bonta IL, Ben-Efraim S. Involvement of inflammatory mediators in macrophage antitumor activity. *J Leuk Biol* 1993; **54**: 613–626.
111. Lee A. Whyte MKB, Haslett C. Inhibition of apoptosis and prolongation of neutrophil functional longevity by inflammatory mediators. *J Leuk Biol* 1993; **54**: 283–288.
112. Hurley JV. Acute Inflammation. 2nd ed. London: Churchill Livingstone. 1983: 109–117.
113. Takeda Y, Watanabe H, Yonehara S, Yamashita T, Saito S, Sendo F. Rapid acceleration of neutrophil apoptosis by tumor necrosis factor-α. *Int Immunol* 1993; **5**: 691–694.
114. Mangan DF, Mergenhagen SE, Wahl SM. Apoptosis in human monocytes: possible role in chronic inflammatory diseases. *J Periodontol* 1993; **64**(suppl 5): 461–466.
115. Henderson S, Rowe M, Gregory C, *et al.* Induction of bcl-2 expression by Epstein–Barr virus latent membrane protein 1 protects infected B cells from programmed cell death. *Cell* 1991; **65**: 1107–1115.
116. Levine B, Huang Q, Isaacs JT, Reed JC, Griffin DE, Hardwick JM. Conversion of lytic to persistent alphavirus infection by the bcl-2 cellular oncogene. *Nature* 1993; **361**: 739–742.
117. Rao L, Dabbas M, Sabbatini P, Hockenbery D, Korsmeyer S, White E. The adenovirus E1A proteins induce apoptosis, which is inhibited by the E1B 19 kDa and bcl-2 proteins. *Proc Natl Acad Sci USA* 1992; **89**: 7742–7746.
118. Jacobson MD, Burne JF, Raff MC. Programmed cell death and bcl-2 protection in the absence of a nucleus. *EMBO J* 1994; **13**: 1899–1910.
119. Terai C, Kornbluth RS, Pauza CD, Richman DD, Carson DA. Apoptosis as a mechanism of cell death in cultured T lymphoblasts acutely infected with HIV-1. *J Clin Invest* 1991; **87**: 1710–1715.
120. Laurent-Crawford AG, Krust B, Muller S, *et al.* The cytopathic effect of HIV is associated with apoptosis. *Virology* 1991; **185**: 829–839.
121. Ameisen JC, Capron A. Cell dysfunction and depletion in AIDS: the programmed cell death hypothesis. *Immunol Today* 1991; **12**: 102–105.
122. Groux H, Torpier G, Montë D, Mouton Y, Capron A, Ameisen JC. Activation-induced death by apoptosis in CD4+ T cells from human immunodeficiency virus-infected asymptomatic individuals. *J Exp Med* 1992; **175**: 331–340.
123. Gougeon ML, Olivier R, Garcia S, *et al.* Mise en evidence d'un processus d'engagement vers la mort cellulaire per apoptose dans les lymphocytes de patients infectés par le VIH. *C R Acad Sci III* 1991; **312**: 529–537.
124. Meyaard L, Otto SA, Jonker RR, Mijnster MJ, Keet RPM, Miedema F. Programmed death of T cells in HIV-1 infection. *Science* 1992; **257**: 217–219.

ACKNOWLEDGEMENTS. The authors are grateful to Sia Timmerman for her excellent secretarial assistance.

**Received 6 September 1994; accepted 15 September 1994**
